# Nutritional Strategies in Prediabetes: A Scoping Review of Recent Evidence

**DOI:** 10.3390/nu12102990

**Published:** 2020-09-29

**Authors:** Jun Wern Yau, Sze Mun Thor, Amutha Ramadas

**Affiliations:** Jeffrey Cheah School of Medicine and Health Sciences, Monash University Malaysia, Bandar Sunway 47500, Malaysia; jwyau2@student.monash.edu (J.W.Y.); smtho21@student.monash.edu (S.M.T.)

**Keywords:** prediabetes, diabetes mellitus, type 2, hyperglycemia, glucose intolerance, diet therapy

## Abstract

Nutritional therapy has been conventionally recommended for people with prediabetes as a method to delay or halt progression to type 2 diabetes. However, recommended nutritional strategies evolve over time. Hence, we performed a scoping review on recently reported nutritional interventions for individuals with prediabetes. Ovid MEDLINE, PubMed, Embase, Scopus, CINAHL and PsycINFO databases were searched to identify relevant research articles published within the past 10 years. Ninety-five articles involving a total of 11,211 participants were included in this review. Nutritional strategies were broadly classified into four groups: low calorie diet, low glycemic index diet, specific foods, and a combination of diet and exercise. The most frequently assessed outcomes were plasma glucose, serum insulin, serum lipid profile, body mass index and body weight. More than 50% of reported interventions resulted in significant improvements in these parameters. Nutritional interventions have demonstrated feasibility and practicality as an effective option for prediabetes management. However, the intervention variability demonstrates the challenges of a ‘one-size-fits-all’ approach. Investigations in genetically diverse populations and objective assessment of progression rate to diabetes are necessary to better comprehend the impact of these nutritional strategies in prediabetes.

## 1. Introduction

The risk of developing type 2 diabetes mellitus (T2DM) increases with existence of one of two precursor states—impaired fasting glucose (IFG) and impaired glucose tolerance (IGT). IFG is defined as a fasting plasma glucose (FPG) concentration of ≥6.1 but <7.0 mmol/L, whereas IGT is defined as an FPG concentration of <7.0 mmol/L and a 2-h post-load plasma glucose concentration of ≥7.8 but <11.1 mmol/L following an oral glucose challenge [1]. A glycated hemoglobin A1c (HbA1c) level of 5.7–6.4% has been included in the existing American Diabetes Association (ADA)’s criteria for high diabetes risk since 2010 [2].

Prediabetes is broadly defined as blood glucose levels above normal but below that of diabetes [3,4]. Prediabetes is typically an umbrella term encompassing IGT, IFG as well as elevated HbA1c levels—all of which are considered substantial risk factors for progression to overt diabetes [3,4]. However, there is much scrutiny surrounding the term prediabetes, partially attributable to its possible reversion to normoglycemia. In addition, prediabetes is not only a risk factor for diabetes, as the risk for diabetes also increases with other conditions such as obesity and hypertension [3]. The necessity of intervention has been also questioned, since not all prediabetic individuals advance to overt diabetes.

According to a recent report by the United States Center for Disease Control and Prevention (CDC), prediabetes awareness remains low at approximately 15% [5]. The importance of studying prediabetes could not be overstated as progression to diabetes entails both macrovascular complications, such as coronary artery disease, peripheral arterial disease, stroke, and microvascular complications, including diabetic retinopathy, nephropathy, and neuropathy [6]. A diagnosis of prediabetes would increase endocrinologist consultations by 78%, leading to possible earlier detection of complications [7].

While it is imperative to prevent diabetes for the sake of precluding future complications, it is also beneficial for reducing the individual and national economic burden in terms of healthcare expenses [6,7]. Furthermore, the diagnosis of diabetes brings about issues with self-perception as well as adverse effects of medications [7]. Therefore, it is of utmost importance that measures are taken to prevent prediabetes from progressing to overt diabetes.

An individualized medical nutrition therapy (MNT) designed by a dietitian or nutritionist should be provided to prediabetic individuals to facilitate achieving treatment aims, as recommended by the 2019 Standards of Medical Care in Diabetes [2]. The ADA also highlight that the MNT is crucial in the overall management plan of diabetes and that healthcare providers need to reassess a diabetic patient’s MNT regularly, especially when there are changes in health status and life stages [8].

Nutritional interventions play a pivotal role in preventing prediabetes from advancing to overt diabetes. The efficacy of such interventions in regulating the progression of prediabetic states to T2DM was underscored in the landmark Diabetes Prevention Program (DPP) [9]. The DPP went on to establish lifestyle modifications, such as dietary change, physical activity, and weight loss, as the ‘gold standard’ for the prevention of T2DM [9]. Nutritional interventions contribute to weight loss by allowing calories to be derived from healthier sources, which appears to be the primary driver to reduce diabetes risk [10]. Moreover, nutritional strategies in prediabetes are also cost-effective tools for reducing the monetary burden with regards to personal as well as public healthcare expenditures. For example, the diabetes cost in the United States was estimated to be approximately USD245 billion in 2012 [11]. The expenditure consisted of direct and indirect medical costs which were USD176 billion and USD69 billion, respectively.

Considering that the major trials in this area were performed at the turn of the 21st century, new evidence is much needed to support their findings. Hence, the aim of this scoping review is to elucidate recently reported nutritional interventions for individuals with prediabetes.

## 2. Materials and Methods

### 2.1. Study Design

We conducted a scoping review to identify nutritional strategies that were studied among adults with prediabetes. A scoping review method was chosen instead of a systematic review, as we aimed to respond to the research question in a comprehensive manner rather than to analyze specific interventions or outcomes.

We utilized the scoping review approach outlined by Arksey and O’Malley [12], Levac and colleagues [13] and the Joanna Briggs Institute [14]. The development of the scoping review protocol was guided by the Preferred Reporting Items for Systematic Reviews and Meta-Analyses (PRISMA) Extension for Scoping Reviews [15]. Ethical approval was not sought as the data were publicly available.

In this review, we defined prediabetes as any or a combination of the following, based on ADA guidelines [2]: IGT, IFG, and elevated HbA1c. Nutritional strategies were defined as any interventions that aimed to change energy intake, macro- or micro-nutrient intakes, specific food or food group, or overall dietary patterns. We included nutritional interventions that fulfilled these criteria and were conducted among adults (18 years and above) regardless of the location, trial design, intervention provider, length of intervention and sample size.

### 2.2. Search Strategy

We conducted a literature search to identify relevant studies in the Ovid MEDLINE, PubMed, Embase, Scopus, CINAHL and PsycINFO databases from 2010 to 2020. This is to ensure the evidence obtained is current.

A search strategy was devised using the keywords and Boolean operators (‘pre-diabetes’ OR ‘impaired glucose tolerance’) AND (diet OR nutrition). Supplementary Table S1 shows the search syntax and strategy for Ovid MEDLINE conducted on 27 April 2020. The syntax and strategy were adapted for the remaining electronic databases.

### 2.3. Study Selection

The study selection process was facilitated using Covidence software [16]. The articles obtained from the databases searches were imported to Covidence where duplicated hits were deleted, publication titles and abstracts were screened and finally, full-text articles reviewed for eligibility based on the review’s inclusion criteria. We did manual hand-searching of the bibliographies of included trials. Two researchers (J.W.Y. and S.M.T.) independently reviewed, discussed, and agreed upon the eligibility of all studies. A third researcher (A.R.) resolved any conflict in agreement between J.W.Y. and S.M.T. Eligible studies were included if the details of the dietary intervention could be extracted, including supplement extracts from food source and prebiotics. Studies were excluded based on several predetermined criteria (Supplementary Table S2).

### 2.4. Data Extraction

Data extraction was conducted using a two-step process. First, we developed a Google Sheet-based template to extract key data extraction variables—country, study design, duration, population under investigation, mean age of participants, intervention and control regimens, measured outcomes and main findings. Subsequently, we reorganized the studies according to setting, focus and outcomes that formed the theme of this review.

## 3. Results

### 3.1. Study Selection and Characteristics

**Figure 1:**
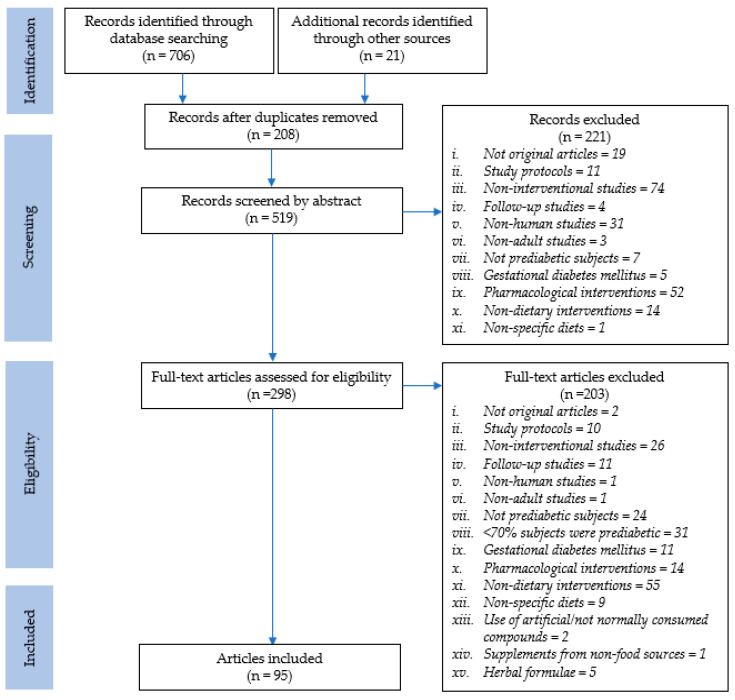
Preferred Reporting Items for Systematic Reviews and Meta-Analyses (PRISMA) flow chart depicting the literature search and study selection process.

A total of 706 records were identified through database searches and 21 records were identified through manual searching. A total of 208 duplicate records were removed and subsequently, the titles and abstracts of 519 records were screened for inclusion. The full texts of 298 articles were reviewed for eligibility, and finally, 95 studies were included in the review (Supplementary Table S3). The literature search and study selection process are summarized in Figure 1.

**Figure 2:**
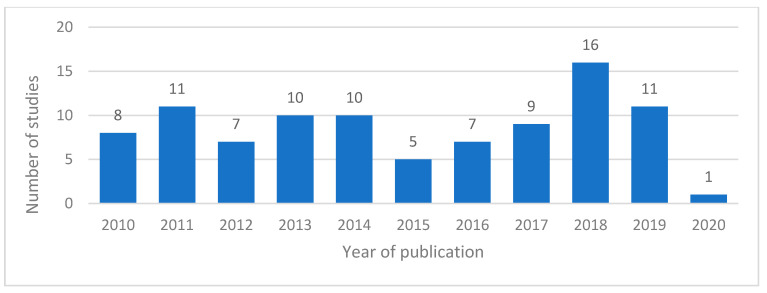
Bar graph showing the number of included studies for each publication year.

The included studies comprised a total of 11,211 participants across 27 countries (Table 1). The Asian population was most studied with 4373 (38.9%) participants. However, United States recorded the highest number of publications with 29 (30.5%) studies. Most studies were randomized-controlled trials (RCTs). Most years have between seven and 11 studies relevant to this review achieving publication, with the exception of 2015, 2018 and 2020 (Figure 2). The small number of studies in the latter could be explained by the inclusion of studies up until the end of April 2020 and not the full year.

### 3.2. Intervention Characteristics

Although 49 studies did not explicitly describe the intervention providers, dietitians or nutritionists provided most of the remaining interventions (Table 2). The recruitment of participants was primarily performed in the community via advertisements, health screening programs and word of mouth. Approximately 15% of study subjects were recruited in general practices and hospitals, respectively. Once recruited, participants were often followed-up in research centers, universities, or healthcare facilities.

ADA definitions of IFG, IGT and elevated HbA1c levels were used by almost all studies to define prediabetes. A total of 48 (50.5%) studies defined prediabetes using a combination of the three criteria. While prediabetes was a criterion for inclusion, overweight or obesity was also a prerequisite for 33 (34.7%) studies.

### 3.3. Nutritional Strategies

**Figure 3:**
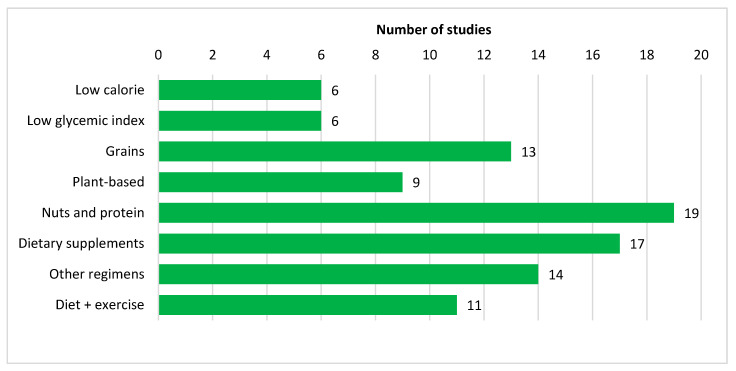
Bar graph showing the number of included studies classified according to interventions.

Nutritional interventions can be broadly divided into four groups: low calorie diet, low glycemic index diet, specific foods, as well as combined diet and physical activity interventions (Figure 3). Supplementary Table S4 provides comprehensive information on the intervention focus of the included studies.

#### 3.3.1. Low Calorie Diet

Six studies [62,67,71,99,102,109] reported low calorie interventions in people with prediabetes, achieved either through restrictions on all food classes or manipulation of a specific food group. Four studies reported low-calorie dietary interventions that were achieved through restrictions on the daily intake of all food classes, with intervention durations lasting from eight weeks to 12 months [62,67,102,109]. Daily energy allowances ranged from 415–1700 kcal, distributed with macronutrient compositions of 41–65% carbohydrates, 12–43.7% protein and 13–30% fat. The studies reported reductions in fasting blood glucose levels, which corresponded to a decrease in insulin resistance in three of the studies [62,102,109]. Measures of anthropometry changed considerably after intervention, with decreases in body weight [67,102,109] and waist circumference [62,67,102]. Interestingly, Christensen et al. compared a low energy diet (810 kcal/day) with a very low energy diet (415–554 kcal/day) and found that the former was preferable owing to lower lean tissue loss and fewer side effects reported [67].

A hypocaloric diet was combined with manipulation in fat intake in two studies [71,99]. These interventions resulted in weight reduction and lower insulin resistance, even in interventions as short as seven weeks [71,99].

#### 3.3.2. Low Glycemic Index Diet

The studies included here are those in which a low glycemic index (GI) diet was specified by the authors or was indicated to be taken by participants. Three studies utilized nonspecific low GI diets as the intervention diet. Hari et al. [74] and Solomon et al. [98] employed a low GI diet (40 arbitrary units) as intervention and a high GI diet (80 arbitrary units) as control, concurrent with a 12-week aerobic exercise training program. These diets were formulated using the 2002 GI tables from Foster-Powell et al. [112] and contained different foods isocaloric to the participants’ individual requirements. Da Costa et al. [101] conversely, reported a low-GI dietary intervention that included a reduction of 25–30% of the total energy intake achieved by incorporating selected plant-based carbohydrate-fiber foods according to the Brazilian food pyramid, in addition to metformin for 16 weeks.

Of the three studies with the nonspecific low GI diet as intervention, two established that insulin sensitivity and peripheral insulin resistance were improved and decreased respectively, whereas nonsignificant differences in insulin sensitivity between low and high GI diet were found in the study by Hari et al. [74,98,101]. Pancreatic β-cell function was successfully preserved by having a low GI diet, ensuring effective glucose metabolism [101]. This was further supported by Solomon et al. who found that postprandial glucose levels improved in a low GI diet while pancreatic β-cell function was impaired in a high GI diet [98]. All three studies demonstrated significant weight loss in low GI diets [74,98,101].

A low GI diet achieved by a combination of dairy, chicken, nuts and whole grains was contrasted against a red meat and refined grain diet in a crossover RCT by Kim et al. [105]. The former proved to be superior to the latter by demonstrating decreased postprandial glucose, insulin and triglyceride responses. It should be noted that, in addition to having lower GI, the glycemic load of the dairy/chicken/nuts/whole grain diet was 42% less than that of the red meat/refined grain diet.

Resistant starch (RS), a low GI carbohydrate, was investigated by two RCTs. In the Korean study by Kwak et al., rice containing RS derived from corn starch, with 6.51g of resistant starch per serving, was served once daily for four weeks [21]. This intervention yielded significant overall improvements to glucose and insulin outcomes compared to the RS-free placebo. However, when a RS-rich diet was compared to a diet rich in fiber in the 12-month study by Dodevska et al., the fiber-rich diet prevailed in achieving better postprandial glycemic control [61]. This RS-rich diet—consumed by subjects under free-living conditions—consisted of plant foods proven to be good RS sources, such as cooked potatoes and beans. Nevertheless, Dodevska et al. demonstrated that body mass index (BMI) and lipid profiles were augmented by the RS diet [61], while Kwak et al. showed that oxidative stress was reduced, leading to better endothelial function [21].

#### 3.3.3. Other Dietary Regimens

Twelve studies reported other dietary strategies studied among people with prediabetes. The Mediterranean diet was studied in two trials, which involved 564 participants [49,106]. Both studies reported no improvement in glucose regulation. In contrast, Roncero-Ramos et al. [49] found that the Mediterranean diet increased the rate of progression to T2DM compared to a low-fat diet. This study also pointed out that both hepatic insulin resistance and HOMA-IR were not improved. Nonetheless, favorable results in lipid profile and BMI were observed in another study [106].

A monounsaturated fatty acid (MUFA)-enriched diet shown to be less proinflammatory compared to a medium-chain saturated fatty acid (SFA) diet [66]. Surprisingly, diets high in MUFA conferred additional benefits of diminishing hepatic fat and improving insulin sensitivity, as a trial by Errazuriz et al. [72] demonstrated. Although this study found no short-term advantage for high-fiber diets, others have shown clear improvements in body weight, blood glucose levels and plasma triglycerides [24,65]. Low advanced glycation end products (L-dAGEs) showed satisfactory results in terms of lipid profile, high-sensitivity C-reactive protein (hs-CRP) level and intima-media thickness, thereby decreasing cardiovascular risk factors [51,66].

A low-energy breakfast [107] and four types of carbohydrates [41] were each investigated in one study and provided positive outcomes. Improvements in glucose metabolism and insulin parameters were observed after consuming a low-energy breakfast [107]. Amongst the four types of carbohydrates (glucose, trehalose, sucrose, isomaltose) examined in the study by van Can et al., sucrose demonstrated the most potential in improving glucose levels, insulin parameters and postprandial inhibition of fat oxidation [41]. 

Food type and proportion may play important roles in determining health outcomes, as suggested by a nonrandomized study by Tippens et al., where consumption of grains, dairy, meat and fat were reduced [90]. This resulted in improved serum lipid profiles and glucose-related parameters including HbA1c, insulin and hs-CRP.

Carnevale et al. [50] examined the effects of extra virgin olive oil and found significant reductions in glucose and triglyceride levels and dipeptidyl-peptidase 4 (DPP4) activity, as well as increases in insulin and glucagon-like peptide-1 (GLP-1). When vinegar was consumed just before meals, it enhanced insulin sensitivity, insulin resistance, glucose metabolism, lipid profile as well as muscle blood flow [63].

While most studies investigated the health effects of food content, two studies [64,86] examined meal patterns, comprising meal frequency and early time-restricted feeding (eTRF). Higher meal frequency (six meals/day) successfully enhanced glucose metabolism and reduced desire to eat compared to the standard three meals/day [64]. As for eTRF, insulin parameters, blood pressure, β-cell function and oxidative stress levels were augmented in prediabetic men [86].

#### 3.3.4. Diet and Physical Activity

Eleven of the reported interventions were not exclusively nutritional, but rather a combination of diet and exercise [22,26,27,29,30,34,38,40,75,84,88]. Overall, diet–exercise interventions demonstrated better results than each of these alone [29,75]. Notably, five studies with a total of 1872 participants examined the effects of increased physical activity with a diet of ~50% carbohydrates, <35% fat and increased fiber [22,26,38,40,88]. These studies discovered significant improvements in diabetic status [22,26] as well as reductions in metabolic syndrome components [38,40,88], even outperforming metformin treatment [38].

A well-balanced diet with plenty of fruit and vegetables, compounded with ample exercise, was supported by large studies as effective strategies for slowing diabetes progression and complications [29,30,34], as was reducing alcohol intake [27,30]. Likewise, caloric and fat restrictions with regular physical activity successfully lowered HbA1c levels, insulin resistance and the risk of advancing to drug-treated diabetes [27,75,84].

#### 3.3.5. Specific Food/Food Groups

##### Grains

Fourteen studies investigated the effect of various types of grains in people with prediabetes [19,23,28,31,32,43,56,57,58,76,77,85,87,105]. Five studies with a total of 298 patients were carried out to investigate the health benefits of a whole grain diet among prediabetic adults [19,23,56,57,77,105]. These studies demonstrated whole grains to be superior to refined grains, with the most notable benefits being better glucose- and insulin-related parameters such as FPG, HbA1c, glucose metabolism, insulin level, insulin sensitivity as well as insulin resistance [19,23,57]. The rate of progression to T2DM and weight loss also demonstrated positive outcomes in favor of the whole grain group [57,77]. Furthermore, plasma inflammatory markers were also reduced in the study by de Mello et al. [56].

Similar outcomes were assessed in two studies investigating the nutritional benefits of flaxseed, as well as two studies comparing the nutritional values of brown rice and white rice [32,76,85,87]. In addition to glucose- and insulin-related parameters, the latter recorded significant weight loss and ameliorations in blood pressure and lipid profiles in the brown rice group [32,87]. Such positive outcomes were also reported in RCTs using quinoa [43], barley flakes [28], cereal fiber [58] and foxtail millets [31] as interventions.

##### Plant-Based Foods

The effect of plant-based foods, notably fruit and vegetables were reported by nine studies [17,18,25,33,82,95,96,100,108]. Berries, which include strawberries, blueberries and cranberries, were the most studied among plant-based interventions [95,96,100]. While an overall improvement in insulin sensitivity was reported, berries showed no effects on BMI, blood pressure, lipid profile, inflammatory markers and glucose metabolism.

Kimchi [17], soybean leaf and banana extracts [18], curcumin [33], natto (Japanese fermented soybeans) and viscous vegetables [25], ultraviolet B (UVB)-treated mushrooms [82], and kiwi [108] were the subjects of one study each and showed overall positive outcomes in prediabetic patients. Glucose metabolism was only shown to be improved significantly in three studies [17,18,33]. Effects on insulin-related parameters were augmented in all plant-based interventions, except kiwi and UVB-treated mushrooms. Other metabolic risk factors such as BMI, blood pressure and lipid profile showed improvement at postintervention in kimchi, natto and viscous vegetables, kiwi and soybean leaf extract studies [17,18,25,108]. Curcumin was found to improve β-cells function and insulin resistance with few adverse effects [33].

##### Nuts and Protein-Based Foods

A significant number of studies (n = 19) investigated the effect of nuts and protein-based foods on prediabetes. Proteins derived from milk and soybeans were the most common interventions in this group, accounting for three studies each. Significant improvements in brachial artery flow mediated dilatation, oxidative stress markers and glucose metabolism, especially postprandial glucose as well as HbA1c, were observed in these participants. However, no evidence supported the benefit of milk proteins to insulin parameters and lipid profiles [60,79,81]. Glucose levels, BMI and insulin parameters were enhanced in soy protein compared to placebo [20,35,59].

High-protein diets (25–30% of daily energy requirements) were compared with diets of lower protein content (15% of daily energy requirements) in two RCTs [93,110]. Interestingly, Stentz et al. [93] found complete remission of prediabetes in the high-protein group. Conversely, two studies describing an intervention with fatty and lean fish and Camelina sativa oil (CSO) discovered little improvement other than better serum lipid profiles for the CSO group [54,55]. Similarly, measurements of renal function after one year of a high protein, low GI diet in the large “PREVIEW” trial did not differ significantly from a moderate protein and GI diet [111].

Nuts are important sources of protein, especially for individuals practicing vegetarianism. The health effects of pistachio in prediabetes were studied extensively in the EPIRDEM trial [44,45,46,47,48]. Consumption of pistachio brought positive genetic changes in DNA damage, diabetes-related circulating miRNA and gene expression of some telomere-associated genes, leading to a healthier genetic profile [44,48]. Pistachio also demonstrated positive correlations with glucose and insulin related parameters, urine metabolites and creatinine levels in prediabetes [44,45,47]. However, except for small LDL particles and non-HDL particles [46], there were no significant improvements in lipid profile and inflammatory risk factors [45].

Wien et al. [89] explored the advantages of almonds and discovered marked improvements in fasting insulin levels, HOMA-IR and HOMA-B scores. The average LDL cholesterol level also showed significant reduction after 16 weeks of intervention with almonds.

##### Dietary Supplements

Nine of 17 studies which investigated various dietary supplements used vitamin D as the intervention [36,37,42,68,69,83,91,92,94]. Most showed an increase in serum 25-hydroxyvitamin D levels but failed to demonstrate improvements in FPG, insulin sensitivity, insulin resistance and lipid status. Results for rates of progression to T2DM [36,91] and β-cell function [42,69,92] in the vitamin D group were particularly confounding, as contradicting outcomes were reported.

Both thiamine [103,104] and L-arginine [52,53] supplements were discussed in two studies each, which found improvements in insulin sensitivity, rate of progression to T2DM and blood pressure.

The effects of inulin [70], chromium [97], galacto-oligosaccharides [39] and betaine [73] supplementation were reported by one study each. None reported any significant positive metabolic effect nor decrement in diabetic risk factors.

### 3.4. Outcomes

**Figure 4:**
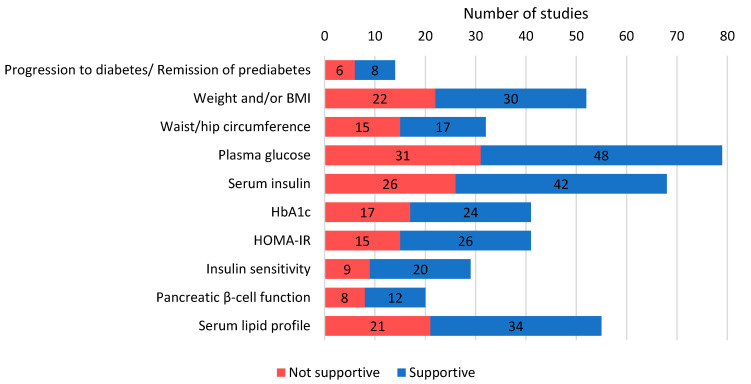
Bar graph showing number of studies reporting selected main outcomes and their proportions that demonstrated statistically significant improvements. Note: HbA1c, glycosylated hemoglobin; HOMA-IR, homeostatic model assessment of insulin resistance.

Overall, we categorized the reported study outcomes into five categories: general outcomes, anthropometry, glucose- and insulin-related parameters, additional parameters and other parameters. In addition to mapping the number of studies to each outcome (Supplementary Table S5), we also recorded and tallied the studies showing significant improvements in individual categories (Figure 4). This was done to substantiate and further elevate the gathered evidence.

#### 3.4.1. General Outcomes

We identified 14 studies assessing the rate of progression to diabetes or remission of prediabetes, which carries prognostic significance, with intervention durations ranging from eight weeks to nine years. Eight studies (57.1%) recorded statistically significant improvements [22,26,27,33,34,36,53,93]. An assessment of performance in physical activity or aerobic fitness, often measured by maximal peak oxygen consumption (VO_2_ max), across 10 studies showed improvement in seven of them (70%) [27,29,40,71,74,98,111].

Among studies with dietetic advice, education or support as the primary intervention, seven studies evaluated participants’ eating behavior or changes in dietary intake [22,27,34,65,88,90,106]. All of them found a preponderance towards healthy eating such as reduced daily caloric, fat and alcohol intake as well as increased fiber consumption.

#### 3.4.2. Anthropometry

Thirty of 52 studies (57.7%) that investigated changes in weight and/or BMI, reported significant improvement postintervention [17,22,24,26,27,29,31,32,33,40,43,58,59,61,65,71,74,77,84,87,88,90,93,98,99,101,102,109,110,111]. Similar proportions of statistically significant findings were observed for the waist and hip circumferences and/or waist-to-hip ratio (17/32 studies, 53.1%) [17,18,24,26,32,33,38,58,61,62,84,87,88,101,102,108,109] as well as systolic and diastolic blood pressure (16/31 studies, 51.6%) [17,31,32,38,40,62,86,87,88,93,98,102,104,108,109,111]. Fat mass, fat-free mass and/or percent body fat were detailed by 22 studies, of which 15 (68.2%) demonstrated positive outcomes [17,18,29,67,69,71,74,77,93,98,101,102,109,110,111].

#### 3.4.3. Glucose- and Insulin-Related Parameters

Understandably, the most assessed parameter was plasma glucose, often interpreted as FPG and/or two-hour postprandial glucose (2hPG) measured via an oral glucose tolerance test (OGTT). Of 79 studies investigating plasma glucose levels, 48 (60.8%) found significant improvements in one or more of the following categories: FPG, 2hPG and area under the postprandial curve (AUC) over variable timeframes up to 180 min [17,18,19,20,21,23,24,25,26,27,28,31,32,33,37,38,40,41,44,49,50,52,57,58,59,60,61,62,64,65,74,76,78,79,81,85,88,90,93,98,102,103,105,107,108,109,110,111]. Likewise, serum insulin levels were measured with similar classifications and recorded a comparable number of significant results (42/68 studies, 61.8%) [17,19,21,23,25,28,33,36,41,50,53,58,59,62,63,64,68,71,73,74,76,77,78,79,81,85,86,87,89,90,92,93,94,95,98,99,101,103,105,107,109,110]. This was followed by HbA1c, with 24 (58.5%) of 41 studies showing markedly reduced levels [17,18,19,23,24,28,29,32,33,38,42,43,58,60,64,67,68,83,84,90,91,92,93,108]. This was also reported by two thirds of studies measuring C-peptide responses [19,24,33,53,58,77,94,98,100,105,107,109].

Among computed values derived from glucose and insulin measurements, the homeostatic model assessment of insulin resistance (HOMA-IR score) was most frequently reported with 41 studies, of which 26 (63.4%) demonstrated significant improvement with intervention [17,18,19,21,23,24,28,31,33,36,44,49,53,58,59,62,76,85,86,87,89,93,101,103,109,110]. A variety of methods were employed by 29 studies to measure insulin sensitivity, namely the Matsuda index, insulin sensitivity index (ISI), quantitative insulin sensitivity check index (QUICKI) and the Cederholm index, and 20 (69.0%) signaled improvement [17,25,27,29,37,49,52,56,58,72,73,74,86,93,94,96,98,99,100,101]. Additionally, significantly better pancreatic β-cell function determined via the HOMA-B score as well as the insulinogenic and disposition indices were identified in 12 of 20 studies (60%) [17,33,42,49,53,56,74,77,86,89,92,101].

#### 3.4.4. Additional Blood Parameters

Many blood components investigated had little to no direct association with glucose yet were equally important in determining overall health and influencing diabetes progression. Analysis revealed serum lipid profile to be the most studied with 34 of 55 trials (61.8%) reporting significant reductions of one or all the following: total cholesterol, low-density lipoprotein (LDL) cholesterol, high-density lipoprotein (HDL) cholesterol and triglycerides [18,25,26,28,31,32,38,40,46,50,51,55,57,61,62,63,65,68,69,73,82,84,86,88,89,90,93,98,99,101,102,105,106,109]. Factoring in free fatty acid [40,41,49,57,74,76] and apolipoprotein levels [28,31,50,51,55,68], a total of 38 of 58 studies (65.5%) found positive results in terms of a serum lipid-related parameter.

This was followed by C-reactive protein (CRP) levels, with 17 studies. Often specified as high-sensitivity CRP (hs-CRP), six (35.3%) reported statistically lower postintervention values [51,56,58,90,98,109]. The same applies with five (31.3%) of 16 studies examining other inflammatory markers such as interleukin 6 (IL-6), IL-8, tumor necrosis factor alpha (TNFα) and oxidized LDL (ox-LDL) [19,31,45,56,93]. Adiponectin [33,93], leptin [31,58,99], ghrelin [77], glucagon-like peptide-1 (GLP-1) [45,50,77,107] and gastric inhibitory polypeptide (GIP) [98] are hormones that play important roles in mediating satiety and various metabolic functions. One sixth to half of the studies exploring these regulatory hormones yielded significantly improved outcomes.

#### 3.4.5. Other Parameters

One of the notable outcome parameters reported in these studies was urinary prostanoids such as prostaglandin F2α, albeit opposite effects in this marker were observed with different interventions [19,21,54]. Measures of endothelial function, commonly through brachial artery flow-mediated dilatation but also via carotid arterial stiffness and muscle blood flow, were performed by 10 studies, of which six (60%) reported significant improvements [52,56,63,78,79,81]. Three studies investigated the effect on satiety and appetite and found considerable differences postintervention that translated to better health outcomes [43,64,86].

#### 3.4.6. Adverse Events

While the previous sections have characterized beneficial outcomes, a description of the undesirable health effects would paint a bigger picture on the practicality of nutritional interventions. Upon analysis (more details in Supplementary Table S3), only ten (10.5%) studies reported adverse events [24,33,35,42,66,67,73,86,92,109]. Perhaps the most significant is the study by Pietraszek et al. [66] which compared diets containing MUFA and medium-chain SFA. Both were found to increase plasma IL-6 levels, indicating that dietary fat-rich diets contain proinflammatory elements.

A constellation of symptoms was experienced by slightly less than half of the subjects from the large PREVIEW study, including constipation, muscle weakness and influenza-like symptoms [109]. Mild gastrointestinal disturbances such as nausea, vomiting, abdominal cramping and transient loose stools were reported by multiple studies investigating different interventions, including betaine supplementation [73], dietary fiber [24], early time-restricted feeding [86], curcumin extract [33] and vitamin D supplementation [42].

In addition to gastrointestinal symptoms, mild vaginal bleeding and breast swelling/pain were observed in some women taking soy proteins in the RCT by Liu et al. [35]. Likewise, Christensen et al. reported significantly higher rates of flatulence, halitosis and cold intolerance in subjects consuming the very low-energy diet than those in the low-energy group [67]. There were 28 adverse events in the study by Mitri et al. but details were not disclosed [92]. In general, no serious adverse events were reported.

## 4. Discussion

Adopting the methodological framework by Arksey and O’Malley [12], this scoping review primarily serves to collate and describe existing literature on nutritional interventions for prediabetes, with the aim of ultimately becoming an instrument of reference for future studies. To our knowledge, this is the first scoping review illustrating the intervention focus, measured outcomes and geographical status of recent clinical trials specifically targeting the prediabetic population.

Majority of the studies employed the ADA definition of prediabetes, which includes IGT, IFG and elevated HbA1c levels. Classification of studies by country indicated that prediabetes is widely studied in four continents, but this does not paint the whole picture. Since T2DM is equally prevalent in both high and low-income countries [113], it follows that prediabetes exerts global prevalence, and charts a rapidly increasing course in all countries [114]. The preponderance towards inclusion of more affluent countries such as the United States can be attributed to several factors, including a more advanced healthcare system. By contrast, developing countries are compelled to address more pressing social and political issues [115], and therefore have smaller budgets for research and development, especially lifestyle interventions that can cost an upwards of USD34.06 per kilogram lost [116].

The age of the study participants varied widely from 20 years to approximately 70 years, with the majority classified in the 50–60 years age group. Prevalence of prediabetes increases with age, as evidenced by the 2013–2016 U.S. National Health and Nutrition Examination Survey which put the estimated percentage of population affected at 46.6% over 65 years, in contrast to 24.3% in the 18–44 years age group [5]. While this justifies the recruitment of patient groups, evidence to link increased prediabetes prevalence in the older age demographic could not be found in the included studies. Given an in-depth analysis of the effect of each intervention on specific age groups is beyond the scope of this review, we certainly could not preclude the potential impact of age on the effectiveness of a particular dietary intervention.

Previous studies have associated age-related processes, such as increase in adiposity, skeletal muscle mitochondrial dysfunction and chronic low-grade inflammation, with insulin resistance [117,118,119]. Nutritional interventions may aid in preventing or delaying prediabetes by optimizing the biomarkers correlated with these deleterious processes. Various nutritional strategies, namely whole grains and high-protein meals, have successfully improved levels of CRP (35.3% of 17 studies) and other proinflammatory markers (31.3% of 16 studies), particularly IL-6 and TNFα. Notably, evidence has shown that elevated levels of these markers of inflammation correspond to significantly increased diabetes risk [120,121], thereby adding to the merits of nutrition therapy.

Intervention strategies applied on prediabetic subjects ranged from specific nutrient supplements to combined diets incorporating an amalgamation of food types. Most studies delineated the exact caloric values and macronutrient compositions of the interventional and control regimens, allowing for future head-to-head comparisons to optimize and refine therapy. Importantly, 63 of 95 studies (66.3%) experimented on one food group or selected food type, rather than combination diets or generic macronutrient-based categories, such as low-fat or low-carbohydrate aliments. This inherently translates to greater variability in focus which further impugns attempts of recommending a ‘one-size-fits-all’ approach to dietary management of prediabetes.

An all-encompassing dietary regimen also lacks feasibility when considering interindividual variability. This phenomenon can be attributed to differences in gut microbiota, genetic polymorphisms for intestinal transport proteins and various host factors such as age, sex and metabolic status, as highlighted by numerous studies in the past [122,123,124]. Interindividual variability may be a major determinant of the contrasting responses of participants observed in the outcomes of similar nutritional interventions. To address this intrinsic aspect of diet-based interventions, we have highlighted the general agreement regarding specific outcome parameters for each intervention.

Interestingly, close to one third of interventions (31/95 studies) were performed via nutritional education, rather than a closed experimental setting with strict observer monitoring (Supplementary Table S3). Commonly administered by a registered dietitian or nutritionist, these education-based interventions involved multiple follow-ups and regular dietary reviews to ensure compliance. The implementation of nutritional education has the potential to not only exert long-term health impact on participants [125], but also reflect real-world feasibility—an aspect not usually seen in noneducational nutritional interventions.

Although it comes as no surprise that most studies investigate parameters pertaining to glucose and insulin, our results revealed that recent trials were more diverse and far-reaching in terms of reporting, with more than 90 different outcomes measured. While measurements of glucose and insulin responses followed standard approaches, a variety of formulae-based methods were employed in the generation of certain outcome values such as pancreatic β-cell function and insulin sensitivity. This underscores the heterogeneity in evaluating a specific parameter, which may differ significantly depending on the chosen tool. However, this review was not powered to detect which methods possess superiority in accuracy and reliability, as other studies have attempted [126,127].

In its 2019 position statement on MNT, the ADA highly recommends that prediabetic patients engage in an intensive lifestyle intervention program akin to the DPP and/or individualized by a registered dietitian or nutritionist. The objectives of such a program include promoting healthy eating habits, setting a moderate-intensity exercise routine to a minimum of 150 minutes weekly, and if required, attain a sustained weight loss of 7–10% [8].

The DPP, a large-scale trial involving 3234 prediabetic individuals, provided the strongest evidence to date regarding the effectiveness of lifestyle intervention in preventing the progression to overt diabetes, with reduced T2DM incidence of 58% at three years [9], 34% at 10 years [128] and 27% at 15 years [129] follow-up. Similar evidence has been reported in other large-scaled trials such as the Finnish Diabetes Prevention Study (FDPS) [130], China Da-Qing Diabetes Prevention Study [131,132], Japanese Diabetes Prevention Trial [133] and Indian Diabetes Prevention Study [134] (Table 3).

After evaluating the studies in this review, we present a few recommendations for future research. First, there was no study regarding the effects of a solely low-fat diet that fit our inclusion criteria. Although numerous studies explored these practices within the general diabetes theme [8], only a small number of low-carbohydrate diets as well as meal patterns were investigated in the prediabetic population. Hence, this represents an important knowledge gap that future researchers are encouraged to fill by designing studies incorporating those diets as interventions, with special attention given to prediabetes.

Genetic variations and ethnic nuances need to be considered to yield evidence for nutritional interventions that are valid, reliable and generalizable. Certain regions of the world that are culturally rich and genetically diverse such as Africa and some parts of South East Asia were underrepresented in the study pool. Therefore, researchers should endeavor to involve subjects from a variety of backgrounds in future trials. We acknowledge that low-income countries may face various situational challenges effectively prohibiting research and development, but these should be systematically overcome as prediabetes is increasingly recognized as an evolving global issue [114]. A multinational, crowdfunded study involving the collaboration of multiple developing countries with limited means to implement independent research can be considered.

Given that merely 14.7% of the study pool included the rate of progression from prediabetes to T2DM, this important outcome should be given precedence, as it is a direct, high-impact measure of intervention effectiveness. Together with glucose- and insulin-related parameters, the significance of these outcomes should be further highlighted in the abstract, regardless of statistically significant changes, to allow readers to easily recognize the key results of the nutritional intervention. Moreover, additional meaningful yet unexplored outcomes such as quality of life and cost efficiency can be included in future studies. It is imperative that translational studies applying nutritional strategies in real clinical practice be developed and implemented to promote further improvements in prediabetic care.

This scoping review has some strengths that give credit to its validity and reliability as a literary map for rapid analysis in this field. First, this review included a large number of RCTs which increases the credibility of the evidence presented. The sample size in this review paper was respectable, with a total number of 11,211 prediabetic adults included. Studies were limited to the previous 10 years (2010–2020) to facilitate the provision of up-to-date information. Furthermore, studies written in languages other than English, such as Chinese and Spanish, were also included, ensuring the capture of a wide study pool and the absence of language bias.

We found that most studies discussed in this review were able to provide nutritional suggestions that were relatively simple to implement and prescribe to prediabetic patients, thus allowing uncomplicated adoption and assimilation in postindustrial countries. Moreover, the wide variety of focuses and outcomes in these studies can provide extensive room for discussion. Here, an overview of this broad field was established—with structured tables to assist rapid search—to emphasize important elements and improve understanding.

Several limitations were identified in our scoping review. Scoping reviews commonly involve an extensive range of scientific literature found through an array of methods including, but not limited to, manual searching, electronic databases, online search engines and gray literature. However, the search method of this review was limited to electronic databases only, possibly resulting in fewer studies considered in this review than would otherwise be the case if other search strategies were also employed. Furthermore, the methodology of the studies in our review was limited to solely human interventional studies, thereby preserving high quality of evidence. Most studies rejected in the first screening were cohort, case-control, epidemiological and animal studies, whereby some may be of relevance or significance. This further diminished the number of studies included in this review.

In addition, large studies such as those listed in Table 3 were excluded because they could not meet the stringent time frame specified in the inclusion criteria, which was executed to ensure recency. Follow-up and secondary analyses of these studies, despite being implemented after 2010, were also eliminated because they could not satisfy the original study criterion. Finally, full appraisal of studies is generally not performed in scoping reviews, leading to the possibility of including studies of questionable quality. Hence, the conclusions in our review were based on the presence, rather than the quality, of studies.

## 5. Conclusions

The prediabetic state represents a golden opportunity for definitive treatment, beyond which overt diabetes assumes a permanent position as a chronic illness, and lifelong pharmacotherapy is warranted. Given the challenges of addressing such an increasingly prevalent condition, the search for a scalable solution capable of reducing the global burden of disease has now arrived on nutrition. Nutritional interventions, by themselves or part of a larger lifestyle modification program, are a cost-saving option for prediabetic management with high feasibility and practicality in its implementation. Numerous dietetic strategies have been carried out across the world with varying degrees of success in preventing or delaying the onset of diabetes, among other outcomes. A comprehensive summary of recent evidence could be a useful instrument in the making of informed decisions by researchers and clinicians alike. This scoping review aspires to achieve such distinguished status by directing future studies to the highly promising prospects of nutritional strategies in prediabetes.

## Figures and Tables

**Table 1:** Distribution of studies according to geographical locations.

Country	Studies	Participants(N)	RCTs	Randomized, Nonplacebo-Controlled Trials	Non-RCTs	Pre–Post Trials	References
Asia	22	4373	19	1	1	1	[17,18,19,20,21,22,23,24,25,26,27,28,29,30,31,32,33,34,35,36,37,38]
Korea	7	1171	6	1			[17,18,19,20,21,22,23]
Japan	4	986	4				[24,25,26,27]
China	4	827	3			1	[28,29,30,31]
Vietnam	1	60			1		[32]
Thailand	1	240	1				[33]
India	1	485	1				[34]
Hong Kong	1	180	1				[35]
Iran	2	207	2				[36,37]
Saudi Arabia	1	217	1				[38]
Europe	31	2951	25	5		1	[39,40,41,42,43,44,45,46,47,48,49,50,51,52,53,54,55,56,57,58,59,60,61,62,63,64,65,66,67,68,69]
The Netherlands	4	330	3	1			[39,40,41,42]
Spain	7	731	6	1			[43,44,45,46,47,48,49]
Italy	4	258	4				[50,51,52,53]
Finland	4	388	4				[54,55,56,57]
Germany	3	180	3				[58,59,60]
Serbia	2	102	2	1			[61,62]
Greece	3	181	2	1		1	[63,64,65]
Denmark	2	226	2	1			[66,67]
Norway	1	511	1				[68]
Sweden	1	44	1				[69]
Americas (North and South)	33	1413	25	5		3	[70,71,72,73,74,75,76,77,78,79,80,81,82,83,84,85,86,87,88,89,90,91,92,93,94,95,96,97,98,99,100,101,102]
United States	29	1255	23	5		1	[70,71,72,73,74,75,76,77,78,79,80,81,82,83,84,85,86,87,88,89,90,91,92,93,94,95,96,97,98]
Canada	2	56	2				[99,100]
Brazil	1	16				1	[101]
Mexico	1	86				1	[102]
Australasia	7	216	4	2		1	[103,104,105,106,107,108]
Australia	5	190	3	2			[103,104,105,106,107]
New Zealand	1	26	1			1	[108]
Multinational	3	2560				3	[109,110,111]

RCT, randomized-controlled trial.

**Table 2:** Intervention characteristics.

Characteristics	Number of Studies	Percentage of Included Studies (%)
Intervention providers		
Physician	11	11.6
Nurse	9	9.5
Dietitian/nutritionist	30	31.6
Pharmacist	5	5.3
Unknown	49	51.6
Study recruitment location		
Primary care clinic	15	15.8
District/tertiary hospital	14	14.7
Community	47	49.5
Unknown	24	25.3
Prediabetes diagnosis criteria		
IFG + IGT + HbA1c	10	10.5
IFG + IGT	30	31.6
IFG + HbA1c	8	8.4
IFG – ADA (FPG 5.6–6.9mmol/L)	26	27.4
IFG – WHO (FPG ≥ 6.1 but <7.0 mmol/L)	1	1.1
IGT (2hPG ≥ 7.8 but <11.1 mmol/L)	15	15.8
HbA1c (5.7–6.4%)	2	2.1
Others (not defined, or did not follow ADA nor WHO definitions)	3	3.2

2hPG, two-hour postprandial glucose; ADA, American Diabetes Association; IFG, impaired fasting glucose; IGT, impaired glucose tolerance; HbA1c, glycosylated hemoglobin; WHO, World Health Organization.

**Table 3:** Lifestyle modifications in past major type 2 diabetes mellitus (T2DM) prevention trials.

Study/Year	Intervention	Main Findings
The Finnish Diabetes Prevention Study (FDPS) [130] 1993–2002	Aim: 5% weight loss Fat < 30% of total energy Saturated fat < 10% of total energy Fiber > 15 g per 1000 kcal Physical activity: 30 min/day	Body weight and diabetes risk were significantly reduced by lifestyle changes in overweight participants with IGT.
U.S. Diabetes Prevention Program (DPP) [9] 1996–1999	Aim: 7% weight loss Fat < 25% of total energy Physical activity: 150 min/week	Lifestyle intervention and metformin significantly decreased the incidence of T2DM in prediabetic participants, with more notable reductions in the former.
The Da-Qing Impaired Glucose Tolerance (IGT) and Diabetes Study [131,132] 1986–1992	Aim: achieve BMI of 23 kg/m^2^ (if ≥25 kg/m^2^) High-carbohydrate diet Low-fat diet Increase physical activity by 12 units/day	Diet and/or exercise interventions reduced the incidence of T2DM in Chinese participants with IGT.
Japanese Diabetes Prevention Trial [133] 1993–1996	Aim: maintain BMI < 22.0 kg/m^2^ Individualized dietary advice Decrease fat intake (<50 g/day), portion size, alcohol intake and eating out Physical activity: 20–40 min/day	Lifestyle intervention successfully reduced body weight and the 4-year cumulative incidence of T2DM in Japanese males with IGT.
Indian Diabetes Prevention Study [134] 2002–2005	Avoid simple sugar and refined carbohydrate Fat < 20 g/day Increase fiber intake Physical activity: 30 min/day	Incidence of T2DM in Asian Indians was significantly reduced in the lifestyle modification and metformin groups, with no additional benefits in the combined group.

BMI, body mass index; IGT, impaired glucose tolerance; T2DM, type 2 diabetes mellitus.

## Supplementary Materials

The following are available online at https://www.mdpi.com/2072-6643/12/10/2990/s1. Table S1: Search strategy employed for Ovid MEDLINE, Table S2: Exclusion criteria for this scoping review, Table S3: Summary of included studies (n = 95), Table S4: Intervention focus of included studies, Table S5: Outcomes of included studies.
