# Supplementary material for: Nutritional Strategies in Prediabetes: A Scoping Review of Recent Evidence
Source: Nutrients. 2020 Sep 29;12(10):2990. doi: 10.3390/nu12102990 (PMC7650618; doi:10.3390/nu12102990)
Supplement: Supplementary file 1 [file nutrients-12-02990-s001.zip › Table S1.pdf]

Table S1. Search strategy employed for Ovid MEDLINE.

No	Query	Results
1	prediabetes.mp or Prediabetic State/	8078
2	impaired glucose tolerance.mp or Glucose Intolerance/	14804
3	1 or 2	21739
4	diet.mp or Diet/	421301
5	nutrition.mp	203310
6	4 or 5	573581
7	3 and 6	3850
8	Limit 7 to "all adults (19 plus years)"	1416
9	Limit 8 to last 10 years	829
10	Limit 9 to clinical trial	286

[a] Comparable strategy were used with other databases

[b] as of 27th April 2020
