# Supplementary material for: Nutritional Strategies in Prediabetes: A Scoping Review of Recent Evidence
Source: Nutrients. 2020 Sep 29;12(10):2990. doi: 10.3390/nu12102990 (PMC7650618; doi:10.3390/nu12102990)
Supplement: Supplementary file 1 [file nutrients-12-02990-s001.zip › Table S2.pdf]

Table S2. Exclusion criteria for this scoping review

**Paper characteristics**
• Not an original article (i.e. review, meta-analysis, book chapter, conference abstracts, etc.)
• Trial protocols and study designs
**Study characteristics**
• Not an interventional study
• Not an original study (follow-up analysis)
• Not human study
• Focus on children and adolescents
• Not prediabetic subjects
• conducted among a mix of diabetic, prediabetic or normoglycemic subjects but have <70% prediabetic cases
• Subjects recruited while they were pregnant / gestational diabetes mellitus
**Intervention characteristics**
• Pharmacological intervention
• Not dietary intervention
• Non-specific diets (i.e. nutritional content not delineated)
• Use of artificial compounds / compounds extracted from plants not normally consumed as a food item
• Supplement extracts from non-food source
• Herbal formulae
