# Supplementary material for: Nutritional Strategies in Prediabetes: A Scoping Review of Recent Evidence
Source: Nutrients. 2020 Sep 29;12(10):2990. doi: 10.3390/nu12102990 (PMC7650618; doi:10.3390/nu12102990)
Supplement: Supplementary file 1 [file nutrients-12-02990-s001.zip › Table S4.pdf]

Table S4. Intervention focus of included studies

No	Focus	Definition	Number of studies	Total sample	References
**Low calorie diet**		Restriction of daily caloric intake to below 1,700 kcal/day for men and 1,400 kcal/day for women (upper limits)			
**1**	Low calorie diet alone		4	2557	[62,67,102,109]
**2**	Low calorie + low fat diet		2	46	[71,99]
**Low glycemic index diet**		Diets with a glycemic index of 55 or below			
**1**	Non-specific		3	70	[74,98,101]
**2**	Dairy/chicken/nuts/whole grain		1	51	[105]
**3**	Resistant starch-rich diet		2	132	[21,61]
**Other dietary regimens**					
**1**	Mediterranean diet	Diet rich in fresh fruits and vegetables, nuts, fish and olive oil	2	564	[49,106]
**2**	MUFA diet	MUFA comprises 28-72% of total energy intake	2	77	[66,72]
**3**	Fibre-rich diet	Dietary fibre intake within WHO recommendations of 27-40g/day	2	156	[24,65]
**4**	L-dAGEs diet	Avoiding food prepared via grilling, broiling, roasting, searing and frying	1	62	[51]
**5**	Low energy breakfast	20% of daily energy intake comes from breakfast	1	13	[107]
**6**	Four types of carbohydrates	Comparison of the metabolic response elicited by glucose, trehalose, sucrose and isomaltose ingestion	1	10	[41]
**7**	Naturopathic whole foods	Reduction in >1 food type (processed and refined grains, dairy, meat and fat)	1	45	[90]
**8**	Additives	Substances used in preparing or garnishing food in order to improve its taste	2	38	[50,63]
**9**	Meal patterns	Consumption of meals based on the time and frequency of intake during the day	2	59	[64,86]

**Diet and physical activity**		A combined lifestyle intervention involving both dietary changes and modulation of physical activity levels			
**1**	Low carbohydrate + low fat + high fibre intake + exercise		6	1987	[22,26,29,38,40,88]
**2**	Low calories + exercise		1	23	[75]
**3**	low calories + low fat + exercise		1	76	[84]
**4**	High vegetable intake and low alcohol and sugar intake + exercise		2	1027	[30,34]
**5**	Low fat intake + low alcohol intake + exercise		1	304	[27]
**Specific Food/ Food Groups**					
**Grains**		Small, hard seeds commonly consumed as staple foods			
**1**	Whole grains diet		5	298	[19,23,56,57,77]
**2**	Flaxseed		2	36	[76,85]
**3**	Brown rice		2	160	[32,87]
**4**	Quinoa		1	29	[43]
**5**	Barley flake		1	100	[28]
**6**	Cereal fibre		1	117	[58]
**7**	Foxtail millet		1	70	[31]
**Plant-based**		Foods directly derived from plants and fungi			
**1**	Berries		3	94	[95,96,100]
**2**	Kimchi		1	21	[17]
**3**	Natto and viscous vegetables		1	11	[25]
**4**	UVB-treated mushroom		1	43	[82]

**5**	Kiwi		1	26	[108]
**6**	Soybean leaf extract		1	45	[18]
**7**	Curcumin		1	240	[33]
**Nuts and protein-based**		Foods that serve as common sources of protein for various populations			
**1**	Milk		3	66	[60,79,81]
**2**	Soy protein		3	264	[20,35,59]
**3**	High protein diet		3	58	[93,110,111]
**4**	Eggs		2	40	[78,80]
**5**	Fatty fish, lean fish and Camelina sativa oil		2	151	[54,55]
**6**	Pistachio		5	240	[44,45,46,47,48]
**7**	Almond		1	65	[89]
**Nutrient supplements**		Products taken orally that contain a dietary ingredient which can be used to supplement a daily diet in hopes of providing a physiological benefit			
**1**	Vitamin D		9	1232	[36,37,42,68,69,83,91,92,94]
**2**	Thiamine		2	24	103,104]
**3**	L-arginine		2	166	[52,53]
**4**	Inulin supplementation		1	18	[70]
**5**	Chromium		1	60	[97]
**6**	Galactooligosaccharides		1	44	[39]
**7**	Betaine		1	27	[73]

L-dAGEs, low advanced glycation end products; MUFA, mono-unsaturated fatty acid; UVB, ultraviolet B; WHO, World Health Organization
