# Supplementary material for: Nutritional Strategies in Prediabetes: A Scoping Review of Recent Evidence
Source: Nutrients. 2020 Sep 29;12(10):2990. doi: 10.3390/nu12102990 (PMC7650618; doi:10.3390/nu12102990)
Supplement: Supplementary file 1 [file nutrients-12-02990-s001.zip › Table S5.pdf]

Table S5. Outcomes of included studies

No	Outcome	Number of studies	Total sample	Studies with significant outcomes		References
				No	%	
**General**						
1	Progression to diabetes / remission of prediabetes	14	3472	8	57.1	[22*,26*,27*,29,33*,34*,36*,53*,55,58,65,67,69,93*]
2	Risk of conversion to diabetes (Diabetes Risk Score, COX proportional hazards regression analysis)	2	538	2	100	[49*,84*]
3	Physical performance / aerobic fitness (VO_2 max) / physical activity	10	939	7	70	[27*,29*,40*,65,71*,74*,88,98*,106,111*]
4	Eating behaviour / Dietary intake	7	1841	7	100	[22*,27*,34*,65*,88*,90*,106*]
**Anthropometry**						
1	Weight and/or BMI	52	7458	30	57.7	[17*,18,19,20,22*,24*,25,26*,27*,29*,31*,32*,33*,35,36,39,40*,43*,51,58*, 59*,61*,65*,67,68,69,71*,73,74*,77*,84*,85,87*,88*,89,90*,91,93*,96,97, 98*,99*,100,101*,102*,103,104,106,108,109*,110*,111*]
2	Waist and hip circumference / waist-to-hip ratio	32	5483	17	53.1	[17*,18*,19,20,24*,26*,27,31,32*,33*,36,38*,58*,61*,62*,65,67,69,73,82, 84*,87*,88*,89,97,99,100,101*,102*,106,108*,109*]
3	Blood pressure	31	6186	16	51.6	[17*,18,19,20,22,26,31*,32*,36,38*,40*,51,58,62*,65,67,68,73,86*,87*, 88*,89,93*,96,97,98*,99,100,102*,104*,108*,109*,111*]
4	Total fat mass, fat-free mass and/or percent body fat	22	3231	15	68.2	[17*,18*,20,24,29*,31,39,58,67*,69*,71*,74*,77*,93*,96,98*,99,101*, 102*,109*,110*,111*]

**Glucose & glucose-related parameters**					
1	Plasma glucose (fasting plasma glucose,2hPG, AUC glucose)	79	8631	48	60.8	[17*,18*,19*,20*,21*,23*,24*,25*,26*,27*,28*,29,31*,32*,33*,35,36,37*,38*,39,40*,41*,42,43,44*,49*,50*,51,52*,55,56,57*,58*,59*,60*,61*,62*,63,64*,65*,67,68,69,71,72,73,74*,76*,77,78*,79*,81*,82,84,85*,86,87,88*,89,90*,91,92,93*,94,95,96,97,98*,99,100,101,102*,103*,105*,107*,108*,109*,110*,111*]
2	HbA1c	41	6066	24	58.5	[17*,18*,19*,20,23*,24*,25,26,28*,29*,32*,33*,37,38*,39,40,42*,43*,51,58*,60*,64*,68*,69,73,82,83*,84*,87,89,90*,91*,92*,93*,94,97,98,101, 106,108*,109*]
3	Serum insulin (fasting insulin, AUC insulin)	68	7107	42	61.8	[17*,18,19*,20,21*,23*,24,25*,27,28*,29,31,33*,35,36*,37,39,41*,42,44,49,50*,52,53*,55,56,57,58*,59*,60,61,62*,63*,64*,68*,69,71*,72,73*,74*,76*,77*,78*,79*,81*,82,85*,86*,87*,89*,90*,91,92*,93*,94*,95*,96,97,98*,99*,100,101*,103*,105*,107*,108,109*,110*]
4	Insulin resistance (HOMA-IR)	41	5899	26	63.4	[17*,18*,19*,21*,23*,24*,25,28*,31*,33*,35,36*,37,39,42,44*,49*,53*,56,57,58*,59*,60,61,62*,64,68,76*,82,85*,86*,87*,89*,91,93*,94,97,101*,103*,109*,110*]
5	Pancreatic β-cell function (HOMA-B, insulinogenic index, disposition index)	20	1922	12	60	[17*,33*,35,37,42*,49*,53*,55,56*,57,69,72,74*,77*,86*,89*,91,92*,94,101*]
6	Insulin sensitivity (Matsuda index, ISI, QUICKI, Cederholm index)	29	2394	20	69.0	[17*,25*,27*,29*,35,37*,39,42,49*,52*,55,56*,57,58*,60,69,72*,73*,74*,86*,91,92,93*,94*,96*,98*,99*,100*,101*]
7	C-peptide	18	3802	12	66.7	[19*,24*,31,33*,45,53*,58*,68,72,73,77*,94*,98*,99,100*,105*,107*,109*]
8	Glucagon concentration	2	505	1	50	[49*,72]
9	Glycated serum protein	1	180	0	0	[35]
10	Proinsulin	1	240	0	0	[33]
11	Glucose effectiveness	2	150	2	100	[58*,74*]
**Other blood parameters**					
**Lipid-related parameters**					
1	Serum lipid profile (total, LDL- and HDL-cholesterol; TAG)	55	7165	34	61.8	[18*,19,20,22,23,25*,26*,28*,29,31*,32*,38*,39,40*,41,45,46*,50*,51*,55*,57*,61*,62*,63*,64,65*,67,68*,69*,72,73*,78,79,81,82*,84*,86*,87,88*,89*,90*,93*,95,96,97,98*,99*,100,101*,102*,104,105*,106*,108,109*]

2	Free fatty acid (including PUFA)	11	1097	6	54.5	[19,20,29,39,40*,41*,49*,57*,74*,76*,99]
3	Apolipoprotein A & B	8	938	6	75	[20,28*,31*,50*,51*,55*,68*,69]
4	Markers of oxidative stress to lipids (Plasma malondialdehyde, plasma 8-iso-PGF2α and arachidonic acid)	8	376	8	100	[19*,23*,78*,79*,80*,81*,86*,87*]
5	Free glycerol	1	44	0	0	[39]
6	Plasma phospholipid fatty acids	1	72	1	100	[54*]
7	Thiobaribituric acid reactive substance	1	11	1	100	[85*]
8	Plasma Lp-PLA2 activity	1	80	1	100	[23*]
**Hormones**						
1	Adiponectin	12	670	2	16.7	[24,25,31,33*,45,66,76,87,93*,99,100,108]
2	Leptin	9	471	3	33.3	[25,31*,39,45,58*,66,87,99*,108]
3	GLP-1	6	224	4	66.7	[31,39,45*,50*,77*,107*]
4	GIP	3	88	1	33.3	[45,77,98*]
5	Ghrelin	2	39	1	50	[77*,108]
6	Cortisol	2	39	0	0	[73,86]
7	IGF-1	1	27	0	0	[73]
8	Parathyroid hormone	4	687	3	75	[68*,69*,82,94*]
9	Cholecystokinin	2	43	2	100	[78*,79*]
10	Peptide YY	2	57	0	0	[39,77]
11	Thyroid-stimulating hormone	1	27	0	0	[73]
12	Fibroblast growth factor 19	1	27	0	0	[73]
**Vitamins**						
1	Vitamin B12	1	27	0	0	[73]

2	Thiamine status, RBC thiamine pyrophosphate	2	24	1	50	[103*,104,]
3	Plasma antioxidants (vitamin C, E, carotenoids)	3	91	3	100	[80*,90*,108*]
4	Serum vitamin D and its metabolites (25OHD,25OHD2,25OHD3)	9	1245	8	88.9	[36*,37*,38*,68*,69*,82,83*,92*,94*]
5	Folate	1	27	0	0	[73]
**Liver-related parameters**						
1	Liver function (bilirubin, plasma ALT, AST, GGT, ALP)	6	2907	6	100	[18*,27*,29*,67*,73*,109*]
2	Clotting factors (platelet count, fibrinogen, tissue factor, PAI-1, vWF)	3	226	2	66.7	[45*,56*,100]
3	Plasma trimethylamine N-oxide and trimethylamine moieties (choline, L-carnitine, betaine, and γ-butyrobetaine)	1	18	0	0	[70]
**Blood tests**						
1	CRP / hsCRP	17	3642	6	35.3	[19,51*,55,56*,58*,66,67,68,76,80,86,90*,96,98*,104,108,109*]
2	Inflammatory markers (IL-6, IL- 8, TNF-α, ox-LDL and others)	16	797	5~~7~~	~~43.8~~31.3	[19*,31*,39,45*,55,56*,66~~*~~,76,80,85,86,87,93*,95~~*~~,96,100]
3	Blood count (hematocrit, leukocyte count)	2	144	1	50	[58*,73]
4	Renal function (creatinine, eGFR, uric acid, BUN, electrolytes, Urea-creatinine ratio)	5	610	2	40	[68,73*,97,103,111*]
5	Serum calcium	6	876	0	0	[36,68,69,73,82,94]

6	Monocyte chemoattractant protein 1	1	32	0	0	[96]
**Enzymes**						
1	DPP-4 activity	1	30	1	100	[50*]
2	Plasma superoxide dismutase activity	1	85	0	0	[21]
3	Peripheral blood mononuclear cells enzyme activity (TNF-α, IL-6, IL-1, IL-1β, Lp-PLA2)	2	179	2	100	[19*,23*]
**Carbohydrates and proteins**						
1	Endogenous secretory soluble receptor for advanced glycation end products	2	116	0	0	[45,51]
2	Fructosamine	3	106	0	0	[25,31,76]
3	Arginine	3	64	3	100	[52*,80*,81*]
4	1,5-Anhydroglucitol	1	11	0	0	[25]
5	Serum Advanced Glycation End products	1	100	1	100	[87*]
6	Plasma methylglyoxal	2	43	1	50	[78,79*
**Genetics**						
1	DNA damage, leucocyte telomere length and gene expression	2	83	2	100	[44*,66*]
2	MicroRNAs	2	91	2	100	[48*,83*]
3	Cyclic guanosine monophosphate level	1	22	1	100	[52*]
**Others**						
1	Nitric oxide and metabolites	3	64	3	100	[52*,80*,81*]

2	Plasma vasoconstrictors (Ang-II and ET-1)	1	20	1	100	[80*]
3	Phenolic bioavailability	1	41	1	100	[100*]
4	E-selectin	1	131	1	100	[56*]
5	Plasma anthocyanins and their bioavailability	1	21	1	100	[95*]
**Urine parameters**						
1	Urinary prostanoids	3	256	3	100	[19*,21*,54*]
2	Urinary metabolites (e.g. hippurate, p-cresol sulfate, cis-aconitate, dimethylamine, U9.365, MHP)	1	39	1	100	[47*]
3	Urine volume, urinary creatinine clearance, urine albumin, microalbumin	2	67	1	50	[97,111*]
4	Urinary ketone bodies	1	192	1	100	[67*]
**More specific parameters**						
**General**						
1	Satiation and fullness degree, appetite	3	88	3	100	[43*,64*,86*]
2	Energy expenditure and substrate oxidation	2	54	1	50	[39,41*]
**Gastrointestinal system-related**						
1	Fecal short-chain fatty acid	1	44	0	0	[39]
2	Fecal microbiota composition	2	70	1	50	[39*,108]
**Hepatorenal system-related**						
1	Hepatic fat content	3	185	2	66.7	[29*,72*,73]

2	Hepatic glucose production and hepatic insulin resistance	1	23	1	100	[75*]
3	Liver radiodensity and volume	1	15	1	100	[99*]
**Cardiovascular system-related**						
1	Intima thickness	1	62	1	100	[51*]
2	Endothelial function (Arterial stiffness / Brachial artery flow-mediated dilation / Muscle blood flow)	10	387	6	60	[51,52*,56*,63*,73,78*,79*,81*,86,97]
3	Endothelial function by number of CFU-EPCs	1	144	1	100	[53*]
4	Left ventricular volume and function	1	15	1	100	[99*]
5	Incidence of microvascular complications	1	542	1	100	[30*]
**Nervous system-related**						
1	Brain reactivity	1	27	0	0	[110]
2	Thermogenic response	1	10	0	0	[41]
**Others**						
1	Bone mineral density	1	2224	1	100	[109*]

* demonstrates significant ($p<0.05$) improvement

25OHD,25-hydroxyvitamin D; 2hPG,2-h postprandial glucose; 8-iso-PGF2α,8-iso-prostaglandin F2α; ALP, alkaline phosphatase; ALT, alanine aminotransferase; AST, aspartate aminotransferase; AUC, area under the curve; Ang-II, angiotensin II; BMI, body mass index; BUN, blood urea nitrogen; CFU-EPCs, colony forming units of endothelial progenitor cells; CRP, C-reactive protein; DNA, deoxyribonucleic acid; DPP-4, dipeptidyl peptidase-4; ET-1, endothelin 1; GGT, gamma-glutamyl transferase; GIP, gastric inhibitory polypeptide; GLP-1, glucagon-like peptide-1; HDL, high-density lipoprotein; HOMA-B, homeostasis model assessment of β-cell function; HOMA-IR, homeostasis model assessment of insulin resistance; HbA1c, glycosylated hemoglobin; IGF-1, insulin-like growth factor 1; IL, interleukin; ISI, insulin sensitivity index; LDL, low-density lipoprotein; Lp-PLA_2, lipoprotein-associated phospholipase A_2; PAI-1, plasminogen activator inhibitor-1; PUFA, polyunsaturated fatty acid; QUICKI, quantitative insulin sensitivity check index; TAG, triacylglyceride; TNF-α, tumor necrosis factor-α; eGFR, estimated glomerular filtration rate; hsCRP, high-sensitivity C-reactive protein; ox-LDL, oxidized low-density lipoprotein; vWF, von Willebrand factor
